# Electrochemical Determination of Nanoparticle Size: Combined Theoretical and Experimental Study for Matrixless Silver Nanoparticles

**DOI:** 10.3390/molecules27082592

**Published:** 2022-04-18

**Authors:** Monika Adamowska, Bartosz Pałuba, Wojciech Hyk

**Affiliations:** 1 Faculty of Chemistry, University of Warsaw, Pasteura 1, PL-02-093 Warsaw, Poland; m.adamowska2@student.uw.edu.pl (M.A.); b.paluba@student.uw.edu.pl (B.P.); 2 Faculty of Chemistry, Biological and Chemical Research Center, University of Warsaw, Żwirki i Wigury 101, PL-02-089 Warsaw, Poland

**Keywords:** silver nanoparticles, chronoamperometry, microelectrode, diffusion coefficient, nanoparticle size

## Abstract

A chronoamperometric procedure for the preparation of silver nanoparticles (AgNPs) in aqueous systems with no extra added stabilizing agents is presented. The uniqueness of the prepared nanoparticle systems was explored by theoretical considerations. The proposed theoretical model predicts the structural parameters of the obtained nanoparticle system. The parameters required for the calculations (the zeta potential, conductivity, and effective diffusion coefficient of ionic silver) are available from independently performed measurements. Chronoamperometry at a microelectrode was employed for the evaluation of the effective diffusion coefficient of ionic silver present in the AgNP solution. The values of AgNP radii predicted by the theoretical model for the selected samples were compared to those obtained by Transmission Electron Microscopy (TEM) and Dynamic Light Scattering (DLS) methods. Because of the high polydispersity of the prepared nanoparticle samples, DLS results were overestimated in comparison to both: the TEM results and some theoretical predictions. By correcting the theoretical predictions by the Debye length, the calculated nanoparticle sizes become comparable (within their expanded uncertainties) to those measured in TEM images, especially for the nanosystems at early stages of their formation via the electrosynthesis process.

## 1. Introduction

Diminishing the size of a metallic sample down to the nanoscale results in the occurrence of many interesting properties. The sample is called a nanomaterial when at least one of its dimensions is smaller than 100 nm. Nowadays, metal nanoparticles of nanoscale size in all three dimensions are broadly examined as very promising substances in technology [1], catalysis [2], environmental science [3], and medicine [4]. Silver nanoparticles (AgNPs) in aqueous solutions, because of their antimicrobial activity [5,6,7,8,9], can be used as pharmaceutical preparations or in agriculture as biostimulants to ease plants’ growth [5,10,11].

There are many approaches to the preparation of metal nanoparticles. These include: chemical methods (e.g., the solvothermal method [12,13], chemical reduction [14,15], the sol-gel process [16]) and physical treatments (e.g., ball milling [17], laser ablation [18]). There is one disadvantage concerning the chemical methods mentioned above—they require the presence of stabilizing agents in solutions, such as polymers or organic surfactants. These substances prevent nanoparticles from agglomerating and enrich the matrix of nanoparticle systems. The latter feature may limit the use of NP materials, especially in matrix-sensitive applications (formulations for medicine, cosmetics, agriculture).

Among many physicochemical characteristics of nanoparticle systems, the size of nanoparticles and their distribution are of crucial importance. There are several instrumental techniques routinely employed for the determination of nanoparticle size. These include Transmission Electron Microscopy (TEM), Dynamic Light Scattering (DLS), Single-Particle ICP-MS (SP-ICP-MS), and UV-Vis spectroscopy. None of these are good enough in terms of the accuracy of nanoparticle characterization. TEM allows one to obtain information about the size and shape of nanostructures but is limited to the inspection of a microportion of the sample, so it is difficult to estimate the size distribution of nanoparticles. DLS is a fast and effective technique to estimate the sizes of nanoparticles, but it does not work well in the case of polydisperse samples. A great advantage of this technique, with the aid of an additional module, is the ability to measure the zeta potential, which is a very important parameter describing the stability of the colloidal system. UV-Vis spectroscopy offers an inexpensive method for the examination of nanoparticle systems thanks to the relation between the radius and the length of absorbed electromagnetic waves. However, the information extracted is rather of a qualitative nature. The SP-ICP-MS method is a very promising approach for determining the average radius and size distribution of nanoparticles present in the sample, but it assumes that all nanoparticles have a spherical shape. Another crucial drawback is that the algorithm applied for SP-ICP-MS measurements treat nanoparticles smaller than 10 nm as free ions in the sample matrix. Moreover, SP-ICP-MS allows one to quantify one element at a time in a single particle mode [19].

In this work, a chronoamperometric synthetic method for the preparation of AgNPs in aqueous solutions without external stabilizing agents is presented. The method proposed does not require the addition of supporting electrolytes and it can be easily adapted for the preparation of nanoparticles of other metals (Au, Pt, Zn, Fe, etc.) in aqueous systems. The prepared AgNPs in ultrapure water are stabilized electrostatically by the ionic species that make up the particle. The absence of other substances (additives) in the synthesized materials significantly simplifies the chemical matrix of the systems. This feature allows the AgNP aqueous solutions to be considered matrixless systems. The uniqueness of the prepared nanoparticle systems was the driving force for the derivation of a theoretical model that would be able to characterize the structure of the matrixless AgNPs. The proposed model combines the transport properties of the ionic species (Ag^+^—ionic species stabilizing the nanoparticle system) and the characteristics of the electrical double layer around spherical nanoparticles (AgNPs). The resulting expression allows one to estimate the average radius of nanoparticles. The parameters required for the calculations (zeta potential, the solution conductivity, and effective diffusion coefficient of ionic silver) are available from independently performed measurements. Chronoamperometry at a microelectrode was employed for the determination of the effective diffusion coefficient of the ionic silver present in the AgNP solution.

The experimentally determined zeta potential combined with the Gouy−Chapman theory has also been successfully employed for the modeling of an effective charge density of nanodiamond systems, as shown by Ge et al. [20].

The values of AgNP radii predicted by the theoretical model for the selected samples were compared to those obtained by the TEM and the DLS methods.

The optimization of the synthetic procedure and theoretical modeling for the production of other metal nanoparticle systems are currently being explored in our lab.

## 2. Materials and Methods

### 2.1. Materials

Ag wire (2 mm in diameter, 99.99%, MBB Szklo, Poland) and ultrapure water (Milli-Q System, water conductivity at 25 °C: 0.055 μS/cm) were the only materials used for the synthesis of AgNPs. High-purity sodium nitrate (99.99%, NaNO_3_, Sigma-Aldrich, Poznan, Poland) was chosen as a supporting electrolyte for voltammetric and chronoamperometric experiments. High-purity silver nitrate (99.9%, AgNO_3_, Sigma-Aldrich) was chosen as an analyte for the construction of the voltammetric calibration curve.

### 2.2. Synthesis of Matrixless AgNPs

The synthesis of silver nanoparticles was conducted in a two-electrode system using an AutoLab, model PGSTAT 128N, potentiostat, controlled via software by a PC. Silver wire was used as anode and a glassy-carbon electrode was used as cathode. The electrochemical cell was filled with 100 mL of ultrapure water and the temperature was set to the desired value (preferably to 15 °C). The cell was water-jacketed. The temperature in the cell was controlled using a refrigerated circulator (Polystat, Cole Parmer, Vernon Hills, IL, USA).

**Figure 1:**
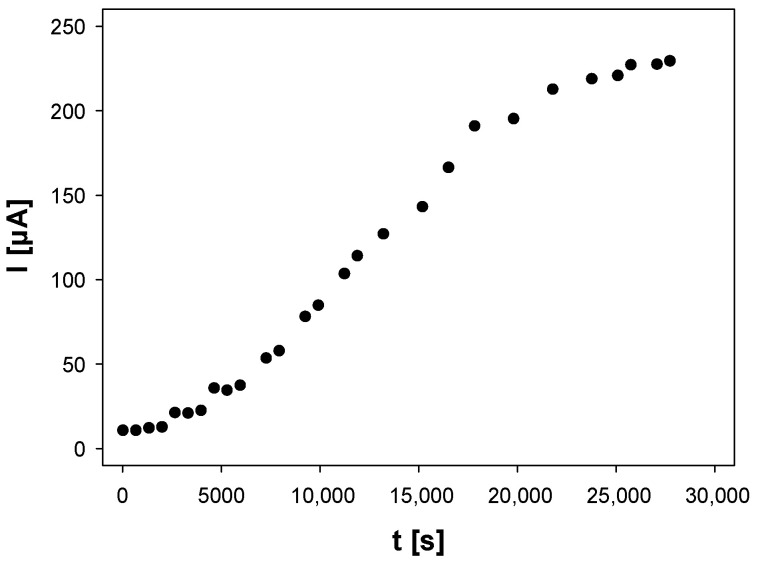
Chronoamperometric curve of silver wire electro-dissolution in the aqueous system. The applied potential: 3.5 V vs. quasi-reference glassy-carbon electrode, T = 15 °C, no supporting electrolyte.

The 8 cm silver electrode immersed in ultrapure water was electrochemically dissolved under chronoamperometric conditions (pulse potential and duration: 3.5 V and 3–8 h, respectively). The electrochemically active area of the silver wire was kept constant for all experiments and was equal to 5 ± 0.2 cm^2^. The experimental conditions (i.e., the temperature of 15 °C and the applied potential), employed for these studies resulted from our previous tests (during which those parameters were variable) and were selected as the most optimal. The exemplary chronoamperogram of metallic silver dissolution in water is presented in Figure 1.

### 2.3. Voltammetric and Chronoamperometric Characterization of AgNPs Systems

The voltammetric and chronoamperometric measurements of silver ion reductions were performed using an AutoLab, model PGSTAT 128N, potentiostat, controlled via software by a PC. All experiments were carried out in the three-electrode system at room temperature. A platinum counter electrode and a platinum quasi-reference electrode, to eliminate a possible leak of electrolyte from the bridge, were used. The latter is especially vital for the systems containing no supporting electrolyte. A 10.5 ± 0.2 (*r_e_* ± u(*r_e_*)) μm in-radius platinum disk microelectrode (nLab, Warsaw, Poland) was used as the working electrode. The surface of the microelectrode was inspected optically with an Olympus model PME 3 microscope. Before each experiment, the microelectrode was polished with aluminum oxide powder of various sizes (down to 0.05 μm) on a wet pad, rinsed with water, and then dried with ethanol. To minimize the electric noise, the electrochemical cell was kept in a grounded aluminum-foil Faraday cage. Before the experiment, the system was degassed with argon for at least 15 min. At least three replicates of voltammograms and chronoamperograms were taken for each experiment under the conditions of either the absence of or excess supporting electrolyte.

The electroreduction of silver cations (i.e., products of the electrogeneration of AgNPs) at a Pt disk microelectrode was used for the determination of both: the concentration and the effective diffusion coefficient of Ag cations.

The concentration of silver ions in NP systems can be determined voltammetrically by employing the calibration curve constructed for the electroreduction of Ag^+^ in aqueous solutions of AgNO_3_ standard samples of well-defined concentrations (in the range of 0.25–1.00 mM), with and without an added supporting electrolyte.

Chronoamperometric measurements at a microelectrode allowed us to determine directly the effective diffusion coefficient of silver cations in the AgNP systems at any level of supporting electrolyte and without knowing the bulk concentration of the cationic species. The theoretical bases, experimental details, and the correctness of the chronoamperometric estimation of the redox species diffusion coefficient at disk microelectrodes were presented in a previous paper [21].

### 2.4. DLS and TEM–EDS Characterization of AgNP Systems

**Figure 2:**
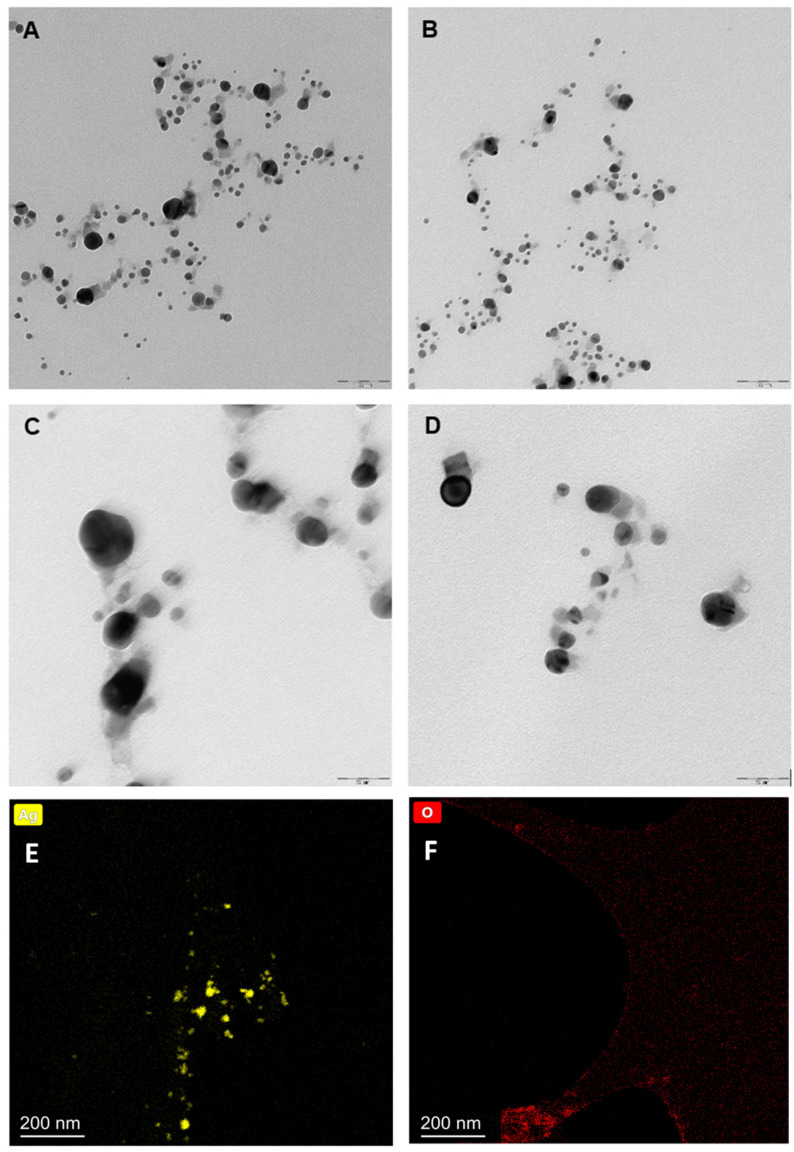
TEM images of matrixless AgNPs. (**A**,**C**) correspond to the systems after a 6-month period of storage, (**B**,**D**) correspond to the freshly prepared systems, and (**E**,**F**) represent EDS elemental mapping for Ag and O for the freshly prepared samples. The scale is 100 nm for the (**A**,**B**) and 50 nm for the (**C**,**D**) images.

Dynamic Light Scattering (DLS) measurements were carried out at room temperature using a Zetasizer instrument (Nano ZS, Malvern Instruments, Malvern, UK) to estimate the average hydrodynamic diameter of the synthesized AgNPs in aqueous solutions. The size and shape of the nanoparticles were also visualized by Transmission Electron Microscopy (TEM) (Libra 120 (Zeiss), Jena, Germany). The exemplary TEM images of AgNP samples (freshly prepared and after a 6-month period of storage) are shown in Figure 2. The images confirm statistically the spherical geometry of the electrosynthesized AgNPs. The chemical composition of the prepared nanosystems was analyzed with Energy Dispersive Spectroscopy (EDS) coupled to the TEM instrument. The results of the elemental mapping for silver and oxygen performed for the freshly prepared samples are shown in Figure 2E and Figure 2F, respectively.

Figure 2F reveals that oxygen, visualized by the EDS mapping procedure, does not overlap with the silver spots visualized in Figure 2E. This means that oxygen is not, in general, chemically bound to silver nanoparticles. In other words, electrosynthesis produces mostly metallic silver nanoparticles. The thin layer of oxides, seen in Figure 2F, might be a consequence of evaporating the aqueous sample during its preparation for TEM measurements.

### 2.5. Theoretical Model

**Figure 3:**
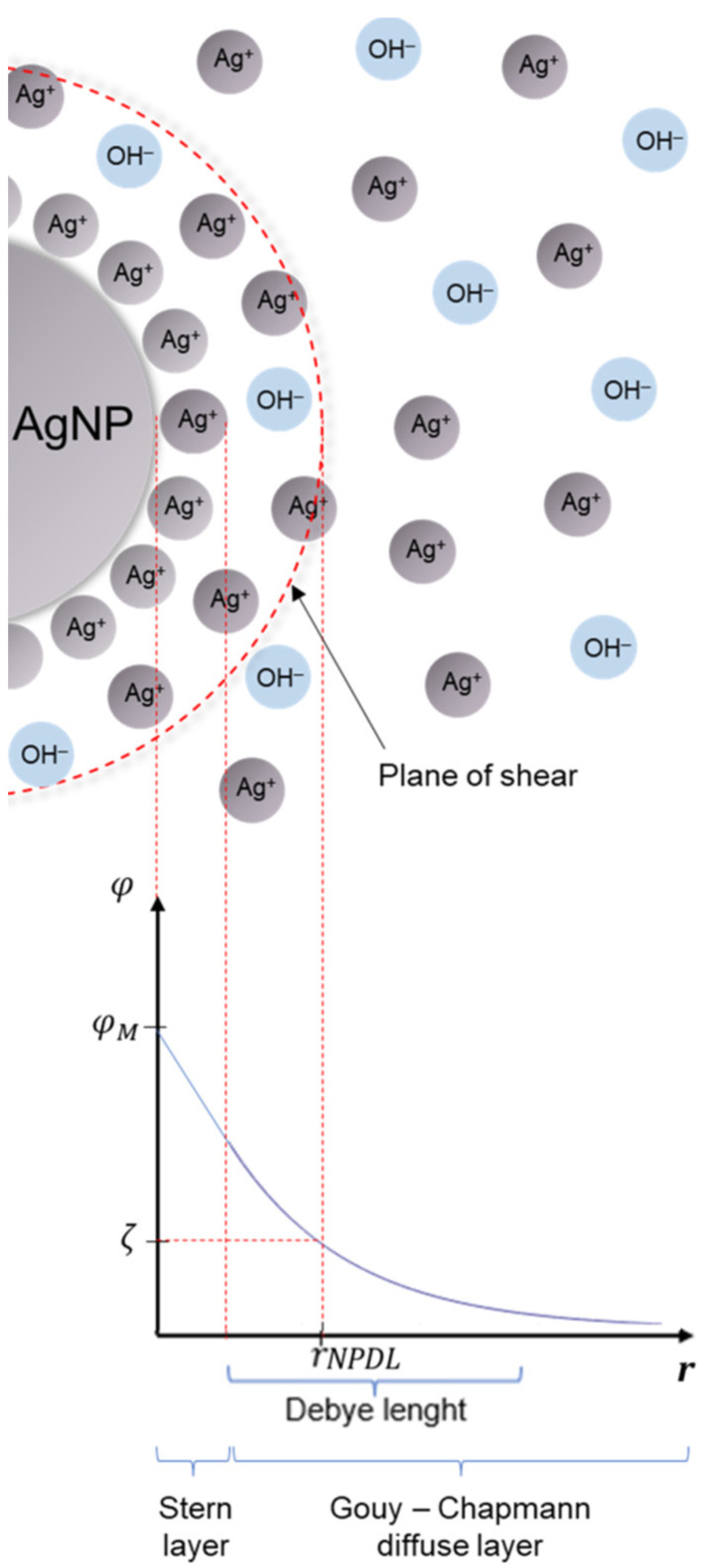
The model of the AgNP system and the crucial parts of the electrical double layer surrounding the nanoparticle. *φ*—electrical potential; *φ*_M_—surface potential of metallic nanoparticle; *ζ*—electrokinetic (zeta) potential; *r_NPDL_*—the *r* distance where zeta potential is measured; *r*—radial distance from the nanoparticle surface.

It was assumed that the double layer around nanoparticles is described by the Stern–Helmholtz model and all nanoparticles are composed only of silver. It is also assumed that the stability of synthesized matrixless AgNPs results from the existence of a thin layer of rigidly bound positive ions (Ag^+^) in Stern’s layer. The diffusive part of the double layer is composed of silver cations, as well as hydroxide anions. It is presumed that broadly examined zeta potential is the electrostatic potential described by the electric charge surrounded by the sphere of the radius equal to the sum of the nanoparticle radius and thickness of the Stern—immobile—layer. To obtain better results, one should also include the plane of shear, which emerges because of the motion of the nanoparticle in the electric field, during the determination of the zeta parameter (Figure 3).

First of all, to evaluate the relation between potential and distance from charged particles, one should consider the Poisson equation in spherical coordinates (because of nanoparticle symmetry):

(1) $$\Delta \varphi =-\frac{\rho }{\varepsilon \varepsilon_{0}\;}\;$$

where *φ* is electrostatic potential [V], *ρ* is volumetric charge density [C m^−3^], *ε*_0_ is vacuum permittivity [C V^−1^ m^−1^], and *ε* is relative permittivity.

Because only the radial variation of *φ* is examined, angular parts depending on *∂* and *φ* angles are not considered, and Equation (1) may be written as:

(2) $$\frac{1}{r^{2}}\;\;\left(\frac{\partial }{\partial r}\;r^{2}\frac{\partial \varphi }{\partial r}\right)=-\frac{\rho }{\varepsilon \varepsilon_{0}}$$

The next step is to find an expression for the volumetric charge density. We propose an equation similar to the Boltzmann distribution (derived from the Nernst–Planck equation):

(3) $$\rho =F\underset{i}{\sum }c_{i}^{b}z_{i}\;exp\left(-\frac{z_{i}F}{RT}\varphi \right)\;$$

where *z_i_* is the charge of the ion *i*, *F* is the Faraday constant (C mol^−1^), *c_i_^b^* is the concentration of ions *i* in bulk, *T* is the temperature (K), and *R* is the gas constant (J mol^−1^ K^−1^).

For relatively small potential values (up to 15 mV), one can use the Taylor expansion of Equation (3) and neglect all its terms except for the linear one. By applying this approximation, one obtains the differential equation in the following form:

(4) $$\frac{1}{r^{2}}\;\;\left(\frac{\partial }{\partial r}\;r^{2}\;\frac{\partial \varphi }{\partial r}\right)=\frac{F^{2}}{\varepsilon \varepsilon_{0}\;RT}\;\underset{i}{\sum }c_{i}^{b}z_{i}^{2}\varphi$$

The introduction of a new variable *χ* = *r* × *φ* into Equation (4) makes it solvable, and the general solution takes the following form:

(5) $$\varphi =A\frac{exp\left(-\lambda^{-1}r\right)}{r}+B\frac{exp\left(\lambda^{-1}r\right)}{r}$$

where *λ*^−1^ is the reciprocal of the Debye length [m^−1^], and *A* and *B* are integration constants.

(6) $$\lambda^{-1}=\sqrt{\frac{F^{2}}{\varepsilon \varepsilon_{0}RT}\;\underset{i}{\sum }c_{i}^{b}z_{i}^{2}}$$

To find a particular solution, the following boundary conditions were set: (1) *φ* → 0 as *r* → ∞ and (2) $\varphi =\frac{Q}{4\pi \varepsilon \varepsilon_{0}r}$ as *λ*^−1^ → 0, where *Q* is electrostatic charge [C] surrounded by the sphere of radius *r* (m)

Then, the final form of the particular solution is given by:

(7) $$\varphi =\frac{Q}{4\pi \varepsilon \varepsilon_{0}r}\;exp\left(-\lambda^{-1}r\right)$$

The electrostatic potential, *φ* in Equation (7), at a certain distance from the nanoparticle surface (denoted by *r_NPDL_* and treated as a sum of the radius of metallic NPs core, *r_core_*, the thickness of the Stern layer, and the thickness of the plane of shear) can be unified with the zeta potential, *ζ* (V), i.e.,:

(8) $$\zeta =\frac{Q}{4\pi \varepsilon \varepsilon_{0}r_{NPDL}}\;exp\left(\lambda^{-1}r_{NPDL}\right)$$

Hence, one can assume that charge *Q* is homogenously distributed inside a sphere surrounding the metallic core, with a certain, constant volumetric density. This density is related to the excessive concentration of one type of ions (in our case cations Ag^+^ with respect to OH^-^ anions) around the core, so the expression for the charge has a form:

(9) $$Q=V\rho =\frac{4}{3}\;\pi r_{NPDL}^{3}\;zFc_{exc}$$

where *V* is sphere volume [m^3^] and *c_exc_* is molar excessive concentration [mol m^−3^].

By combining two Equations (8) and (9), an expression that relates zeta potential to the double-layer size of nanoparticle and the excessive concentration of silver ions is obtained:

(10) $$\zeta =\frac{zFc_{exc}r_{NPDL}^{2}}{3\varepsilon \varepsilon_{0}}\exp\left(-\lambda^{-1}r_{NPDL}\right)$$

The determination of the excessive ionic concentration is the most problematic part of the derivation process. We found that it can be related to the mobility of ionic species. Due to the presence of metallic nanoparticles in a solution and because of their interactions with surrounding ions, it is obvious that the closer to the nanoparticles the ions are, the slower their motion is. Thus, the number and the size of nanoparticles indirectly control the magnitude of ionic species diffusion coefficient. The determination of the latter parameter may employ, e.g., the chronoamperometric response of the microelectrode. It should be emphasized that the electrochemical approach leads to the estimation of the effective diffusion coefficient, which is resultant of the mobilities of both: free silver ions and immobilized silver ions bound electrostatically to nanoparticle surfaces. Therefore, one can estimate the effective diffusion coefficient as a weighted average involving coefficients that quantify the fractions of free silver ions and silver ions enclosed in the nanoparticle double layers.

(11) $$D_{eff}=\frac{\left(D_{Ag^{+}}\cdot c_{free}+D_{NP}\cdot c_{exc}\right)}{c_{free}+c_{exc}}$$

where *D_eff_* is the effective diffusion coefficient (m^2^ s^−1^), *D_Ag+_* is the diffusion coefficient of freely transported silver ions (m^2^ s^−1^), *D_NP_* is the diffusion coefficient of silver nanoparticles with immobilized ions (m^2^ s^−1^), *c_free_* is the concentration of free silver ions (mol m^−3^), and *c_exc_* represents excessive ion concentration bound to the nanoparticles (mol m^−3^).

It is assumed that the concentration of hydroxide ions on the nanoparticle surface is negligible, and the sum of *c_free_* and *c_exc_* represents the total concentration of silver ions, *c_tot_*.

The nanoparticle diffusion coefficient can be estimated by the Stokes–Einstein equation:

(12) $$D_{NP}≅\frac{k_{B}T}{6\pi \eta r_{NPDL}}$$

*k_B_* is the Boltzmann constant (J K^−1^), *T* is the temperature (K), and *η* is the dynamic viscosity of the AgNP aqueous solution approximated by the viscosity of pure water (Pa s).

By inserting Equations (10) and (12) into Equation (11) and making some tedious rearrangements, one obtains an implicit relation for *r_NPDL_* in the following form:

(13) $$0=c_{free}\left(D_{eff}-D_{Ag^{+}}\right)\cdot r_{NPDL}^{3}+\left(\frac{3\varepsilon \varepsilon_{0}\zeta D_{eff}}{zF}\cdot r_{NPDL}-\frac{\varepsilon \varepsilon_{0}\zeta k_{B}T}{2\pi \eta zF}\right)\cdot \exp\left(\lambda^{-1}r_{NPDL}\right)$$

Solving Equation (13) for *r_NPDL_* requires experimental knowledge of the zeta potential, *ζ*, the effective diffusion coefficient of ionic species, *D_eff_*, and the concentration of free Ag^+^ ions, *c_free_*.

The total concentration of silver ions can be extracted from either conductometry or steady-state voltammetry at microelectrodes. The conductivity of the solution is simply connected to the concentration of all ions by the following expression:

(14) $$\kappa =\underset{i}{\sum }z_{i}Fc_{i}u_{i}$$

where *κ* is the conductivity of the AgNP aqueous solution (S m^−1^) and *u_i_* is ion mobility (m^2^ V^−1^ s^−1^), which is directly proportional to its diffusion coefficient ($u_{i}=\frac{D_{i}Z_{i}F}{RT}$).

In our system, we have only silver and hydroxide ions, and because of electroneutrality, both types of ions have the same concentrations, *c_tot_*. Hence, because nanoparticles affect both silver and hydroxide ions, one should calculate mobilities using the effective diffusion coefficient. In the calculations, it was assumed that the *D_eff_* for OH^−^ ions is decreased by the same factor as it is for silver ions. By linking all these facts together, one obtains the following expression for *c_tot_*:

(15) $$c_{tot}=\frac{\kappa RT}{F^{2}\left(D_{eff,\;\;\;Ag^{+}}+D_{eff,\;\;\;OH^{-}}\right)}\;$$

**Figure 4:**
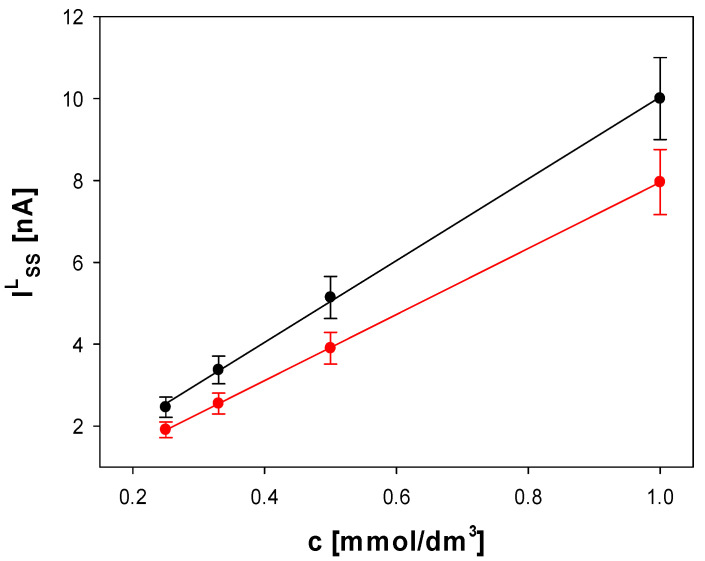
Calibration curves constructed for the voltammetric electroreduction of Ag^+^ at a Pt disk microelectrode in aqueous solutions of AgNO_3_ standard samples (without supporting electrolyte—black symbols, and with excess supporting electrolyte (NaNO_3_, 0.1 M)—red symbols). Cyclic voltammetric curves were recorded at the scan rate of 10 mV/s at room temperature in the potential range: 0 to −0.3 V vs. quasi-reference Pt electrode. The error bars represent standard deviations calculated for the data series of 3–5 replicate recordings of steady-state limiting currents.

Alternatively, the *c_tot_* parameter (as an approximated sum of *c_free_* and *c_exc_*) can be determined voltammetrically by employing the calibration curve constructed for the electroreduction of Ag^+^ in aqueous solutions of AgNO_3_ standard samples of well-defined concentrations (i.e., $I_{ss}^{L}=ac_{Ag^{+}}+b$, where *a* and *b* are regression coefficients of the linear calibration curve for the Ag^+^ concentration range of 0.25–1.00 mM and *I_ss_^L^* is the steady-state limiting microelectrode response for the Ag^+^ reduction) either with or without added supporting electrolyte (Figure 4). The expression for *c_tot_* takes the following form:

(16) $$c_{tot}=\frac{\;{\bar{I}}_{ss}^{L}-b}{a}$$

where ${\bar{I}}_{ss}^{L}$ is the mean steady-state limiting current of the Ag^+^ reduction at the Pt disk microelectrode recorded for the AgNPs system.

The calibration curves presented in Figure 4 are shifted to each other because they were recorded under different mass transport conditions. Under the conditions of supporting electrolyte absence, the migrational contribution to the mass transport makes the faradaic current of the electroreduction of silver cations increase with respect to the purely diffusional conditions (excess supporting electrolyte) [22].

Having determined all necessary parameters, it is possible to find numerically the solutions to Equation (13), i.e., the estimates of the AgNP radius *r_NPDL_* (the sum of the radius of metallic NPs core, *r_core_*, the thickness of the Stern layer, and the thickness of the plane of shear). The numerical calculations were performed using a program written in Python Ver. 3.8 (Wilmington, DE, USA).

## 3. Results and Discussion

The AgNP systems were electrochemically synthesized under the diffusional and migrational conditions by employing the chronoamperometric technique. The electrode processes that occur at silver anode and glassy carbon cathode are represented by the following equations:

Anode:

$${\mathrm{Ag}}^{0}\to {\mathrm{Ag}}^{+}+e^{-}\;2H_{2}O\to 4H^{+}+O_{2}+4e^{-}$$

Cathode:

$${\mathrm{Ag}}^{+}+e^{-}\to {\mathrm{Ag}}^{0}\to \mathrm{AgNPs}\;2H^{+}+2e^{-}\to H_{2}$$

Silver wire (anode) is oxidized to produce silver cations. Glassy carbon cathode reduces silver cationic species to form both the thin deposit of metallic silver on the electrode and to initiate the nucleation process. The latter is probably enhanced by hydrogen (from the reduction of H^+^ ions at the cathode)—a strong reducing agent for silver cations present in the cathode vicinity.

The chronoamperometric electrooxidation of the uncharged substrate (silver electrode material) in the system with no deliberately added supporting electrolyte was extensively studied in our group [23,24]. The theoretical model derived for the migrational chronoamperometry and the experimental results obtained may explain chronoamperometric behavior for the electrosynthesis of AgNPs (Figure 1). The current intensity increases as the conductivity of the system (ionic support) increases to reach the steady-state level. According to the theoretical model published [23], the progress in the electrode process makes the ohmic potential drop diminish and, as a consequence, the true working electrode potential approaches the magnitude of the applied potential. This is a direct reason for the gradual increase in the faradaic current intensity of the silver electrooxidation.

The generated products of the silver electrode electrooxidation were visualized by using TEM. As shown in Figure 2, AgNPs in the obtained samples are of different sizes (3–45 nm in radius). Moreover, the TEM images confirm that electrogenerated AgNPs do not form aggregates even over six months after synthesis. Most of the nanoparticles have a spherical shape, but there do happen to be some of a cubical shape as well. The EDS elemental mapping confirmed the metallic nature of the silver nanoparticles in the prepared samples (Figure 2E,F).

**Figure 5:**
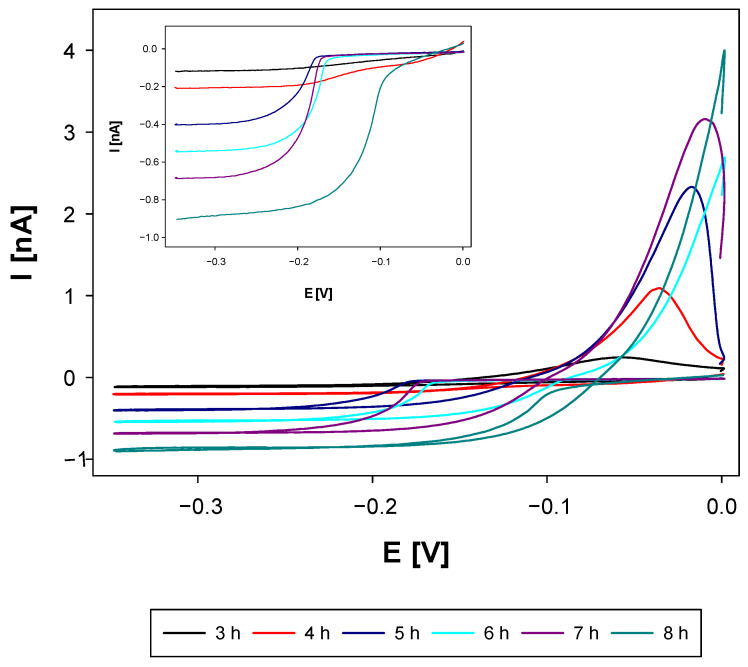
Cyclic voltammograms at Pt disk microelectrode of Ag^+^/Ag reduction/oxidation processes recorded for AgNPs samples taken from electrosynthesis at certain times (3–8 h). Supporting electrolyte: NaNO_3_ (0.1 M), scan rate of 10 mV/s, room temperature, deoxygenation with Ar (15 min). Inset shows reduction voltammetric waves.

According to the model devised (Figure 3), the electrogenerated silver cations stabilize the nanoparticle system. On the other hand, their presence allows one to characterize voltammetrically and chronoamperometrically the prepared samples. The voltammetric responses of the disk Pt microelectrode due to the reduction of Ag cations under the conditions of supporting electrolyte excess are presented in Figure 5.

Figure 5 demonstrates the progress of the NPs synthesis in terms of the amount of electrogenerated Ag^+^ species. It can be seen that the height of the voltammetric plateau of silver ion reduction increases as the synthesis progresses. The longer the synthesis, the more silver ions are generated and a higher probability of silver nucleation is expected. The latter leads to the increased contribution of larger NP sizes in their distribution.

The well-defined voltammetric signals recorded for the prepared AgNP systems gave us a chance for the estimation of the total concentration of silver cations with relatively good accuracy. The voltammetric procedure for Ag^+^ quantification required calibration using the reference samples of the Ag^+^ analyte. The calibration curves were constructed for the reference samples of AgNO_3_ by employing the unweighted linear regression method, and the obtained regression equations are as follows: $I_{ss}^{L}=8.071\left(\pm 0.026\right)c_{Ag^{+}}-0.117\left(\pm 0.016\right)$ (with correlation coefficient of 0.99998) and $I_{ss}^{L}=9.98\left(\pm 0.17\right)c_{Ag^{+}}+0.051\left(\pm 0.099\right)$ (with correlation coefficient of 0.9994) for the excess and absence of a supporting electrolyte, respectively (Figure 4). The quantification results for Ag^+^ in the AgNP samples were calculated on the basis of Equation (16).

**Figure 6:**
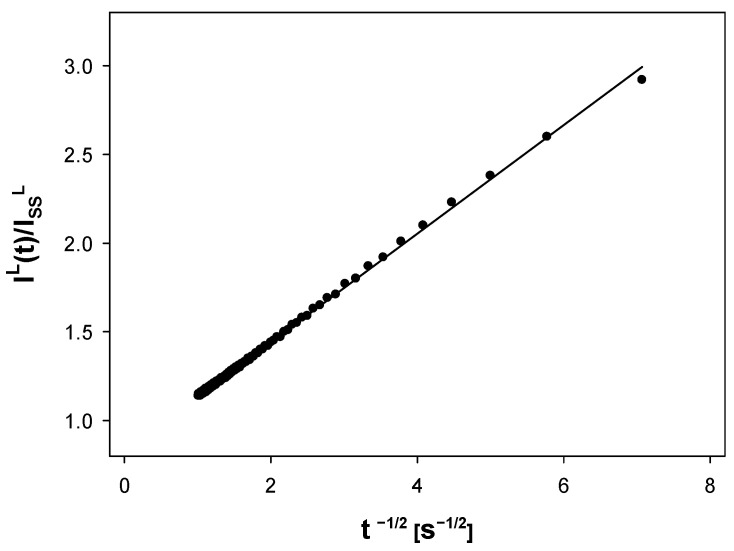
Linearized chronoamperoemtric response of Ag^+^ reduction at Pt disk microelectrode for the AgNP system obtained after 8 h of synthesis. Applied potential: −0.30 V vs. Pt quasi-reference electrode, supporting electrolyte: NaNO_3_ (0.1 M), room temperature.

The prepared samples of AgNPs were also explored chronoamperometrically to test the potential possibility of the estimation of electroactive species diffusion coefficient. Figure 6 demonstrates graphically the idea of the procedure employed for the estimation of the apparent diffusion coefficient of silver cations from chronoamperometric measurements. The data presented in this Figure were generated for the sample obtained after the 8 h anodic dissolution of silver wire. The key function in this approach is expressed by the following equation:

(17) $$\frac{I^{L}\left(t\right)}{I_{ss}^{L}}=\frac{r_{e}}{\sqrt{\pi D_{eff}}}\cdot \frac{1}{\sqrt{t}}+1$$

where *t* denotes the experimental time (s), *I^L^*(*t*) is the limiting current recorded at time *t* (A), *I_ss_^L^* is the steady-state limiting current (the plateau height) (A), *r_e_* is the radius of the disk microelectrode (m), and *D_eff_* is the effective diffusion coefficient (m^2^ s^−1^).

The applicability of this approach for disk microelectrodes was analyzed in detail in our previous work [21]. The regression analysis of the model Equation (17) (where the normalized chronoamperometric current represents the dependent variable while the independent variable is given by a reciprocal of the square root of time (*t*)) and the rearrangement of the obtained regression parameters leads to the estimation of the effective Ag^+^ diffusivity.

**Figure 7:**
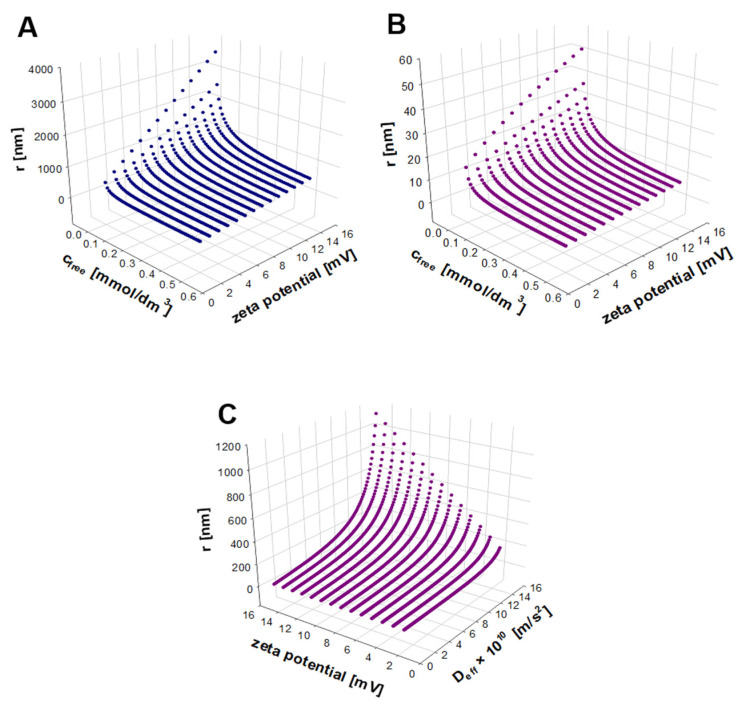
Three-dimensional plots of simulation results (solutions of Equation (13)). The AgNPs’ averaged radii were calculated as a function of zeta potential and the total concentration of ionic species for two extreme values of the Ag^+^ effective diffusion coefficient, *D_eff_*: 1.5 × 10^−9^ m^2^ s^−1^ (**A**), 8 × 10^−11^ m^2^ s^−1^ (**B**), and as a function of the zeta potential and Ag^+^ effective diffusion coefficient for the selected total concentration of Ag^+^ of 0.1 mM (**C**).

To understand better the nature of the produced AgNP systems, a theoretical model that relates the mobility of silver cations accumulated on the surface and in the vicinity of AgNPs with the nanoparticle size was developed. The model is represented by Equation (13). According to this equation *c_tot_*, *D_eff_*, and *ζ* are the key parameters that determine the resulting *r_NPDL_* values. To test the predictive capability of the developed model, numerical calculations were performed for *c_tot_* ranged from 0.01 to 1 mol∙m^−3^, and for *ζ* ranged from 2 to 32 mV. Two extreme values of the Ag^+^ effective diffusion coefficient were taken for simulations: 8 × 10^−11^ m^2^∙s^−1^ and 1.5 × 10^−9^ m^2^∙s^−1^. The obtained simulation results are presented in a form of 3D plots (Figure 7).

The analysis of the dependencies presented in Figure 7A,B reveals several interesting observations for the model matrixless AgNP systems. Firstly, it can be seen that the magnitude of zeta potential has a significant impact on the size of silver nanoparticles, especially at the lowest total concentrations of ionic species. It is seen that the magnitude of the nanoparticle size is positively correlated with the value of zeta potential. It is known that the higher the absolute value of zeta potential is, the smaller the affinity of nanoparticles to form agglomerates. In other words, repulsive forces between nanoparticles make them more distant. This may imply that double layers of nanoparticles overlap to a significantly smaller extent and a larger fraction of ionic species may be freely transported. As an indirect consequence, the effective diffusion coefficient for ionic species approaches its limiting value at infinite dilution. On the other hand, low concentrations of free and bound silver ions may lead to lowering the energetic barrier for nanoparticles to undergo collisions that result in the formation of agglomerates. This observation cannot be supported by the present study because we have no information on the kinetics of the agglomeration process.

It is also seen (Figure 7A–C) that for the same values of zeta potential and total ionic species concentration, larger effective diffusion coefficients are determined for the systems composed of bigger nanoparticles. Assuming the same amount of silver that is used up for the formation of nanoparticles, the increase in the volume of a nanosphere results in the decrease in the number of nanoparticles and, consequently, in the decrease in the active surface for the electrostatic binding of cationic species. Hence, a lower number of larger nanoparticles bind effectively fewer ions to their surfaces, and that is why a larger fraction of free ions occurs. The latter makes the effective diffusion coefficient closer to the value found at infinite dilution.

The theoretical predictions and all experimental characteristics of the synthesized AgNPs systems are summarized in Table 1.

The data collected in Table 1 present the physicochemical characteristics of two AgNP systems synthesized for 8 h under chronoamperometric conditions. The first AgNP system (I) was used to study the time-dependence of the AgNPs’ characteristics by analyzing samples taken from the electrochemical cell every hour. The quantitative information appeared after the third hour. It can be seen for the AgNP system (I) that the magnitudes of the total concentration of ionic silver and its effective diffusion coefficient increase as the chronoamperometric experiment progresses. This is a direct consequence of the increase in the intensity of generating silver cations due to the anodic dissolution of silver wire under the conditions of increased solution conductivity. The values of the total concentration of ionic species were determined independently by using conductance and voltammetry measurements. The obtained results agreed well (at a 95% confidence level), thus the numbers listed in Table 1 represent averaged determinations obtained by these two approaches. Moreover, the longer the synthesis, the more stable nanoparticle systems were obtained according to the zeta potential measurements.

The differences observed for the parameters characterizing the two 8 h AgNP systems (I and II) indicate the strong sensitivity of the final results to the experimental conditions, especially at the initial stage of the experiment. The conditions that alter the final characteristics of AgNP systems may include the unrepeatable conditioning of the working electrode (uncontrolled defects and the existence of oxide deposits) and the presence of undetermined impurities that may act as nucleation seeds, etc.

Table 1 contains the information on the average sizes of the synthesized AgNP systems obtained experimentally (by using DLS (*r_DLS_*) and TEM (*r_TEM_*) techniques) and theoretically (*r_NPDL_*) by employing the proposed model described in Section 2.5.

The DLS technique determines the hydrodynamic radii of nanoparticles and it is rather dedicated to the examination of monodispersive samples. Because of this, *r_DLS_* values are most often overestimated. TEM is the most relevant technique to examine our AgNPs systems because it gives us a chance to detect the shapes and sizes of the cores of nanoparticles present in the solution. Therefore, it is reasonable to treat TEM findings as reference values for alternative approaches. The values of AgNPs’ radii calculated on the basis of our model are overestimated by the inclusion of some part of the electrical double layer. This makes the theoretical predictions greater than those measured by TEM. By subtracting the Debye lengths from the *r_NPDL_* values, one is able to estimate the radius of the nanoparticle metallic core (*r_core_*). The calculated *r_core_* values become statistically consistent with TEM determinations within the expanded uncertainty of *r_core_* at the early stages of the AgNPs’ formation (3–5 h samples). In the process of quantifying the expanded uncertainty, the rules of the propagation of standard uncertainties for implicitly defined measurand (Equation (13)) were employed. The magnitude of the measurand (either *r_NPDL_* or *r_core_*) depends on values of the experimentally determined parameters (i.e., input data: zeta potential, the effective diffusion coefficient, and the total concentration of ionic species extracted from either the solution conductivity or the voltammetric response of a microelectrode). Thus, total variabilities in magnitudes of the experimental parameters (expressed by their relative standard uncertainties) contribute to the combined uncertainty of the measurand. The statistical analysis of repeated measurements of the input parameters revealed that the relative standard uncertainties in their values ranged from 7 to 28%. As a result, the expanded uncertainty of AgNPs radii ranged from 14 to 56%.

## 4. Conclusions

The developed electroanalytical synthetic method was optimized for the preparation of AgNPs in aqueous solutions. No external stabilizing agents were required. The method is easy to implement and can be adapted for the production of nanoparticle systems of other metals. However, the generated nanoparticle systems are characterized by a wide range of nanoparticle sizes from a few nanometers to even micrometers. Because of the high polydispersity of the prepared nanoparticle samples and the presence of the electric double layer around a nanoparticle, the DLS-determined AgNP sizes are, in general, overestimated in comparison to both the TEM results and some theoretical predictions. The theoretical model confirmed that the generated matrixless AgNPs appear to be more stable at a higher radius.

The proposed theoretical model has several limitations. First of all, the radius of the nanoparticle includes also a part of the electric double layer because its determination employs zeta potential. Because of the motion of nanoparticles during zeta potential measurements, it is difficult to estimate how much bigger the nanoparticle radius is in comparison to the metallic core itself. To reduce the overestimation of the predicted nanoparticle radius, one might correct the calculated values by the Debye length (which is an estimate of the thickness of the diffusive part of the double layer), but it should be remembered that the zeta potential is located closer to the metallic core. It means that the value of the difference between the calculated radius *r_NPDL_*, from Equation (13), and the Debye length may underestimate the nanoparticle radius. For very small nanoparticles, this may even generate negative numbers. To overcome these limitations, one should find a more adequate parameter than zeta potential or find a way to estimate the distance from the metallic core surface to the plane of shear better than by the Debye length. Secondly, the model does not include any information about the number of nanoparticles in the solution. This information might be very helpful for a deeper interpretation of the predictions derived on the basis of the proposed model. Therefore, the model would require the inclusion of a certain size distribution.

The last problem concerns the assumed structure of the ionic atmosphere around the metallic core (Figure 3), which is related to the zeta potential—a key parameter for modeling. Zeta potential measurements in our nanosystems are ambiguous—in some cases, the averaged results are either positive or negative or even 0 mV. It may suggest that in one system there are nanoparticles composed of the same silver core but with different ionic surroundings.

## Figures and Tables

**Table 1:** Experimentally determined—and theoretically predicted—characteristics of the synthesized AgNPs systems. The dataset contains the time of synthesis, the average concentration of free silver ions (*c_free_*) from conductivity and voltammetry measurements, the effective diffusion coefficient (*D_eff_*), the zeta potential (ζ), the radius of nanoparticles estimated by TEM (*r_TEM_*), the radius of nanoparticles measured by DLS (*r_DLS_*), the theoretical radius of nanoparticles (*r_NPDL_*, Equation (13)), and the estimated radius of nanoparticle cores (*r_core_*) (i.e., *r_NPDL_* values corrected by the corresponding Debye lengths). The parameters were obtained for two AgNP systems (I—freshly prepared, II—after a 6-month storage).

AgNPs System	Time [h]	*c_free_* [mol∙m^−3^]	*D_eff_*∙10^9^ [m^2^∙s^−1^]	ζ [mV]	*r_TEM_* [nm]	*r_DLS_* [nm]	*r_NPDL_* [nm]	*r_core_* [nm]
I	3	0.0347	0.834	−2.0	-	-	50.1	-
	4	0.0478	0.967	−2.6	-	103.7	61.5	18.2
	5	0.0734	1.043	−5.91	-	127.1	110.3	74.8
	6	0.2178 *	0.448 *	−9.96	-	128.7		
	7	0.1279	1.139	−19.2	-	75.8	294.0	267.1
	8	0.1383	1.155	−20.3	≤40	130.4	314.0	288.1
II	8	0.1445	1.072	−19.0	≤50	45.0	195.2	173.9

* outlying results.

## Data Availability

Not applicable.
